# Dismissed for drinking without drinking: confirmed auto-brewery syndrome in a law-enforcement officer – a case report

**DOI:** 10.1097/MS9.0000000000005716

**Published:** 2026-10-22

**Authors:** Soumyadipto Biswas Arko, Syeda Nazifa Reza, Silvia Monjur Tumpa, Deep Chowdhury

**Affiliations:** a Department of Internal Medicine, Dhaka Medical College Hospital, Dhaka, Bangladesh; b Department of Internal Medicine, Dhaka National Medical College and Hospital, Dhaka, Bangladesh; c Department of Healthcare Administration, University of the Potomac, Washington, DC, USA

**Keywords:** alcohol-use disorder mimic, auto-brewery syndrome, Candida albicans, case report, endogenous ethanol production, gut fermentation syndrome, medicolegal consequences, Saccharomyces cerevisiae

## Abstract

**Introduction:**

Auto-brewery syndrome, or gut fermentation syndrome, is a rare and underrecognized disorder in which gastrointestinal microorganisms ferment ingested carbohydrates into ethanol, causing intoxication without alcohol consumption. Because its clinical presentation mimics alcohol use disorder, affected patients may experience stigma, disbelief, occupational harm, and medicolegal consequences.

**Case details:**

We report a case of confirmed auto-brewery syndrome (ABS) in a law-enforcement officer who developed episodes of dizziness, slurred speech, ataxia, confusion, drowsiness, altered behavior, brain fog, mood changes, hangover-like symptoms, and alcoholic breath odor. Despite denial of alcohol intake, corroborated by his wife, colleagues, friends, and extended family, he was suspected of alcoholism and dismissed from service. During symptomatic episodes, serum ethanol levels were elevated at 62, 106, and 113 mg/dL, with paired breath-alcohol positivity. Repeat testing under chain-of-custody procedures reproduced the same pattern. Urine toxicology was negative; neurologic differentials were excluded; and hepatic encephalopathy was unlikely, with normal liver function tests, international normalized ratio, albumin, and ammonia. A supervised carbohydrate challenge performed in a controlled setting reproduced ethanol elevations and symptoms. Endoscopic cultures grew *Candida albicans* and *Saccharomyces cerevisiae*, while *Klebsiella pneumoniae* was not isolated.

**Discussion:**

This case demonstrates the diagnostic complexity of ABS and emphasizes the importance of correlating symptoms with ethanol measurements, supervised provocation testing, microbiologic evidence, and exclusion of toxicologic, neurologic, hepatic, and laboratory-artifact mimics.

**Conclusion:**

ABS should be considered in patients with recurrent intoxication-like episodes and unexplained ethanol positivity despite credible abstinence. Early recognition is essential to prevent misdiagnosis, stigma, occupational loss, and medico-legal harm.

## Introduction

Auto-brewery syndrome (ABS), or gut fermentation syndrome, is a rare and probably underdiagnosed disorder in which intestinal microorganisms ferment ingested carbohydrates into ethanol, producing objective alcohol intoxication without exogenous alcohol intake^[^1–4^]^. Its true prevalence and incidence remain unknown; the literature is dominated by case reports, and a 2021 systematic review identified only 17 published case reports comprising 20 patients, underscoring both rarity and likely diagnostic under-recognition^[^1^]^. ABS occupies a uniquely confusing space among gastroenterology, microbiology, toxicology, neurology, psychiatry, and forensic medicine because patients present with the phenotype of alcohol intoxication – slurred speech, ataxia, altered behavior, drowsiness, impaired cognition, and alcoholic breath – while denying drinking^[^2–4^]^. Reported organisms include *Saccharomyces cerevisiae* and *Candida* species, with *Candida albicans* described in early classic cases and *S. cerevisiae* repeatedly implicated in later well-documented reports^[^5–7^]^. The diagnosis becomes strongest when recurrent symptoms correlate with elevated blood or breath ethanol, covert alcohol exposure is excluded, a supervised carbohydrate challenge reproduces ethanol elevation, and cultures identify plausible fermenting organisms^[^2–4^]^. Importantly, potentially fermentative fungi may also be present in individuals without ABS; therefore, culture positivity should be interpreted as supportive rather than independently causal^[^8^]^. We report a confirmed ABS case with gastric isolation of *C. albicans* and *S. cerevisiae*, reproducible ethanol elevations, a positive supervised carbohydrate challenge, negative toxicology, exclusion of neurologic and hepatic mimics, sustained post-treatment normalization of carbohydrate challenges, and severe occupational and medicolegal consequences.

HIGHLIGHTS

- Auto-brewery syndrome can mimic alcohol use disorder, with objectively positive ethanol levels.
- A supervised carbohydrate challenge confirmed endogenous ethanol production.
- Serum ethanol levels were reproducibly elevated to 62, 106, and 113 mg/dL.
- Endoscopic cultures grew *Candida albicans* and *Saccharomyces cerevisiae*.
- Misdiagnosis caused major occupational harm to a law-enforcement officer.

## Case presentation

A middle-aged male law-enforcement officer presented with recurrent episodes of unexplained intoxication-like symptoms that had progressively affected his social, occupational, and psychological well-being. During these episodes, he developed dizziness, slurred speech, unsteady gait, ataxia, confusion, altered behavior, drowsiness, “brain fog,” mood changes, hangover-like symptoms, and a fruity/alcoholic odor on his breath. Because of the recurrent clinical appearance of alcohol intoxication and positive alcohol test results, he was suspected of alcohol use at his workplace and was eventually dismissed from his job. The patient repeatedly and consistently denied alcohol intake. His wife, household members, colleagues, friends, and extended family strongly corroborated that he did not consume alcohol, making the occupational accusation particularly distressing for him.

During symptomatic episodes, objective alcohol testing demonstrated elevated ethanol levels. Blood ethanol concentration was documented at 62 mg/dL on one occasion, while two subsequent assessments showed higher values of 106 mg/dL and 113 mg/dL, corresponding to ethanol levels in the clinically significant intoxication-range. Paired breath alcohol testing was also positive. Because covert alcohol intake and laboratory error were important concerns, repeat testing was performed using proper chain-of-custody procedures and serum ethanol analysis at a certified laboratory. The same pattern of unexplained ethanol positivity was reproduced, reducing the likelihood of sample contamination, laboratory artifact, or unreliable testing.

A broad diagnostic evaluation was performed to exclude alternative causes of recurrent altered sensorium and intoxication-like behavior. Urine toxicology screening was negative, arguing against sedative, recreational drug, or medication-related intoxication. Neurological differential diagnoses, including cerebrovascular events, seizure-related episodes, cerebellar pathology, vestibular disorders, and other central nervous system causes were excluded through appropriate clinical and investigational assessment. Hepatic encephalopathy was also considered unlikely because liver function tests, international normalized ratio, serum albumin, and serum ammonia were within normal limits. There was no biochemical evidence to suggest liver failure as an explanation for his altered behavior.

Given the recurrent intoxication phenotype, objectively elevated blood and breath alcohol concentrations, strong collateral confirmation of abstinence, and the exclusion of common mimics, ABS was suspected. The patient subsequently underwent supervised carbohydrate challenge testing in a controlled setting with no access to alcohol. Following carbohydrate administration, serial monitoring showed a rise in ethanol levels with a recurrence of intoxication-like features, confirming endogenous ethanol production. This positive provocation test provided strong functional evidence for ABS rather than hidden alcohol ingestion.

To investigate a potential gastrointestinal source of ethanol production, an upper gastrointestinal endoscopy was performed. Gastric aspirate was obtained and submitted for bacterial and fungal culture. The fungal culture on Sabouraud dextrose agar yielded *C. albicans* and *S. cerevisiae*, whereas *Klebsiella pneumoniae* was not isolated. A gastric mucosal biopsy was also obtained during the procedure. Because *C. albicans* and *S. cerevisiae* may occur as gastrointestinal commensals or transient colonizers in individuals without ABS, these culture findings were interpreted as supportive rather than independently diagnostic. Their clinical relevance was assessed in the context of reproducible systemic ethanol elevations and the positive supervised carbohydrate challenge.

Based on recurrent alcohol-intoxication symptoms, reproducibly elevated blood ethanol concentrations of 62, 106, and 113 mg/dL, paired breath-alcohol positivity, a positive supervised carbohydrate challenge, supportive evidence from gastric isolation of *C. albicans* and *S. cerevisiae*, and exclusion of toxicologic, neurologic, hepatic, and laboratory-artifact explanations, the patient was diagnosed with ABS. The patient was treated with fluconazole 150 mg orally once daily for 21 days together with a low-carbohydrate diet. He was subsequently reviewed at 3-week intervals for 6 months. A supervised carbohydrate challenge was repeated at every follow-up visit and remained normal, with no pathologic ethanol response was demonstrated. Follow-up fungal cultures at 6 months were negative. This longitudinal normalization following therapy provided additional support for the diagnosis and treatment response. The case was notable not only for its diagnostic complexity but also for its severe medicolegal and occupational consequences, when a law-enforcement professional was dismissed for presumed alcoholism despite objective evidence of endogenous ethanol production.

## Discussion

This case highlights why ABS is both biologically fascinating and clinically treacherous. The core mechanism is deceptively simple: fermentable carbohydrates enter a dysbiotic gastrointestinal ecosystem, ethanol-producing microorganisms metabolize sugars through glycolysis to pyruvate and subsequently to acetaldehyde and ethanol, and gastrointestinal ethanol production may exceed the host’s capacity for first-pass and systemic metabolic clearance^[^3,4,9^]^. In most individuals, endogenous ethanol production is minimal and clinically irrelevant; ABS becomes clinically important when microbial abundance and metabolic activity, substrate availability, gastrointestinal transit, mucosal ecology, absorption, and host clearance interact to permit intoxication-level systemic ethanol concentrations^[^3,4,9,10^]^. Thus, ABS is better conceptualized as a functional microbial-metabolic imbalance than as the simple presence of a particular organism. Antibiotic exposure, high-carbohydrate intake, bowel surgery, short bowel syndrome, Crohn disease, pseudo-obstruction, diabetes, obesity, metabolic dysfunction-associated steatotic liver disease, and small intestinal bacterial overgrowth have all been discussed as permissive settings, although otherwise healthy patients have also been reported^[^4,11,12^]^.

The microbiologic findings in this case require cautious interpretation. Upper gastrointestinal endoscopy yielded a gastric aspirate in which fungal culture on Sabouraud dextrose agar identified *C. albicans* and *S. cerevisiae; K. pneumoniae* was not isolated, and a gastric mucosal biopsy was also obtained. *C. albicans* was central to early descriptions of recurrent endogenous intoxication, while *S. cerevisiae* has also been implicated in carbohydrate-challenge-confirmed ABS^[^5,6^]^. A reported case treated with micafungin demonstrated *S. cerevisiae* in stool and *Candida* species in upper gastrointestinal samples, illustrating that potentially fermentative fungi can occur at different gastrointestinal sites^[^13^]^. However, *Candida* and *Saccharomyces* may also be detected in people without ABS, and culture positivity alone cannot establish causality^[^8^]^. In the present case, the microbiologic findings were therefore regarded as supportive. The stronger evidence for disease was functional and longitudinal: repeated systemic ethanol elevations, a positive controlled carbohydrate provocation, and sustained normalization of repeat challenges after antifungal and dietary treatment.

The accumulation of clinically significant ethanol despite hepatic first-pass metabolism does not necessarily imply a missing hepatic enzyme. Ethanol produced within the gastrointestinal tract is absorbed into the portal circulation and is metabolized predominantly through alcohol dehydrogenase and aldehyde dehydrogenase pathways. First-pass extraction is incomplete, and hepatic ethanol elimination has finite metabolic capacity. When intraluminal ethanol production and absorption occur rapidly or continuously enough to exceed metabolic clearance, measurable systemic ethanol concentrations can develop even in the absence of overt liver disease^[^9^]^. In this patient, normal liver function tests, INR, albumin, and ammonia argued against hepatic failure or hepatic encephalopathy. However, alcohol dehydrogenase and aldehyde dehydrogenase activity or genetic polymorphisms were not specifically assessed; interindividual variation in these pathways could theoretically modify systemic exposure and merits further study.

ABS is confusing because nearly every visible feature points toward alcohol use disorder. The patient may look intoxicated, smell intoxicated, have objectively measurable breath alcohol concentration (BrAC)/blood alcohol concentration (BAC) levels, and continue to deny drinking. Clinicians, employers, law-enforcement systems, and even family members may therefore default to an assumption of concealed alcohol consumption. The diagnostic trap is that denial of drinking can occur in alcohol use disorder, while in ABS the same denial may be entirely accurate. The way out is not a credibility judgment but reproducibility: observed abstinence, controlled carbohydrate challenge, serial BAC/BrAC measurements, chain-of-custody ethanol testing, toxicology screening, and appropriate microbiologic or molecular assessment^[^2–4^]^. Our patient’s ethanol values of 62, 106, and 113 mg/dL were reproducibly abnormal, and repeated results obtained with paired BrAC measurements and proper chain-of-custody procedures made analytical artifact substantially less likely.

The differential diagnosis must be explicitly addressed because ABS is a diagnosis requiring positive proof and exclusion. Mimics include covert alcohol ingestion, laboratory contamination, specimen mix-up, post-sampling fermentation, sedative or recreational drug intoxication, diabetic ketoacidosis or ketosis with fruity breath, hypoglycemia, electrolyte disturbance, hepatic encephalopathy, stroke or transient ischemic attack, seizure or postictal state, cerebellar disease, vestibular disorders, encephalitis, Wernicke encephalopathy, psychiatric disease, and *D*-lactic acidosis in short-bowel states^[^3,4^]^. Urinary or bladder fermentation syndrome is another important modern distinction: it can generate urinary ethanol, particularly with glycosuria and *C. glabrata*, but typically lacks systemic intoxication because bladder ethanol does not readily result in blood intoxication^[^14^]^. In contrast, this patient had elevated blood and breath ethanol with neurologic symptoms, supporting gut rather than urinary fermentation.

The medicolegal dimension is not peripheral – it is central. ABS has been discussed in drunk-driving defenses, abstinence-monitoring failures, transplant- and addiction-program testing, criminal justice, and occupational evaluations^[^3,14–16^]^. For a law-enforcement officer, an unexplained positive alcohol test is not merely a laboratory abnormality; it can affect credibility, employment, licensing, fitness for weapon-related or other safety-sensitive duties, family trust, and future income. This case, therefore, illustrates how a rare microbial-metabolic disorder may be misclassified as misconduct when objective ethanol positivity is interpreted without consideration of endogenous production. At the same time, ABS does not make intoxication physiologically harmless or automatically excuse unsafe conduct: endogenously produced ethanol can impair psychomotor function irrespective of its source. Patients with active disease should, therefore, avoid driving, handling weapons or machinery, and other safety-sensitive activities until clinical and biochemical control is established. Rigorous documentation – including observed abstinence, serial measurements, controlled provocation when appropriate, and chain-of-custody procedures – is essential before attributing positive ethanol results either to ABS or to covert drinking.

Treatment is multimodal and should be individualized. Acute high ethanol levels require standard stabilization and appropriate safety precautions. Definitive therapy generally combines reduction of fermentable substrate through a low-carbohydrate or carbohydrate-controlled diet, avoidance of unnecessary antibiotics, culture-guided antifungal therapy when fungi are implicated, and longitudinal monitoring^[^2–4^]^. Reported antifungals include fluconazole, nystatin, itraconazole, and micafungin; resistant or recurrent disease may require susceptibility-guided escalation and specialist input^[^13,17^]^. In our patient, fluconazole, 150 mg orally once daily for 21 days, together with a low-carbohydrate diet, was followed by normal carbohydrate challenges at every 3-week follow-up over 6 months, and negative fungal cultures at 6 months. Probiotics are frequently used with the aim of restoring microbial balance, although evidence for specific preparations remains limited^[^4^]^. Modern management increasingly considers restoration of the microbial ecosystem rather than eradication of a single organism. Fecal microbiota transplantation (FMT) has been reported in refractory long-standing ABS, and contemporary observational work using metagenomic and metabolomic techniques has demonstrated disease-associated microbial ethanol-production pathways^[^10,18^]^. FMT and other microbiome-directed therapies remain investigational rather than established first-line treatment.

Whether the same fungi return after treatment as commensals remains uncertain. Complete and permanent gastrointestinal eradication of *Candida* or *Saccharomyces* is not necessarily required for remission because these organisms may occur in individuals without ABS^[^8^]^. They could theoretically persist or recolonize without causing recurrent disease if the ecological conditions that permit excessive fermentation are no longer present. Accordingly, treatment success is better defined by resolution of pathological ethanol generation than by permanent fungal sterility alone. In this patient, fungal cultures were negative at 6 months and serial carbohydrate challenges remained normal throughout follow-up. Longer longitudinal observation would be required to determine whether later recolonization occurs without recurrence of abnormal fermentation.

Detailed modern microbiome or mycobiome profiling was not performed in this patient. Conventional culture identifies cultivable organisms but cannot quantify organism burden, resolve strain-level ethanol-producing capacity, or characterize wider bacterial-fungal ecological interactions. Future cases would benefit from quantitative polymerase chain reaction (qPCR), fungal internal transcribed spacer sequencing, 16S rRNA sequencing, shotgun metagenomics, metabolomics, and direct ex-vivo ethanol-production assays. Multivariate community analyses such as principal-component analysis or principal coordinates analysis could then evaluate whether active disease, remission, and post-treatment states have distinct microbial signatures. Recent observational work has already used longitudinal metagenomic, fungal sequencing, metabolomic, and functional fermentation approaches in ABS^[^10^]^. Such methods could help distinguish incidental commensal colonization from metabolically active, disease-associated organisms and may eventually support more standardized diagnostic criteria and individualized, microbiome-directed therapy.

The principal strengths of this case are repeated symptomatic ethanol elevations on separate occasions, paired breath-alcohol positivity, repeat chain-of-custody testing, a positive supervised carbohydrate challenge, broad exclusion of major mimics, specific upper gastrointestinal sampling, and 6 months of serial post-treatment provocation testing. Important limitations include the absence of strain-specific in-vitro ethanol-production assays, quantitative fungal burden assessment, detailed microbiome/mycobiome profiling, and direct alcohol dehydrogenase (ADH) and aldehyde dehydrogenase (ALDH) functional or genetic assessment. These limitations prevent attribution of organism-specific causality solely from culture results, but the functional and longitudinal evidence strongly supports endogenous ethanol production consistent with ABS.

## Conclusion

This case represents confirmed ABS supported by recurrent intoxication-like episodes, reproducible blood ethanol elevations of 62, 106, and 113 mg/dL, paired breath-alcohol positivity, and a positive supervised carbohydrate challenge. Upper gastrointestinal endoscopy with gastric aspirate culture on Sabouraud dextrose agar identified *C. albicans* and *S. cerevisiae*, while *K. pneumoniae* was not isolated; these fungal findings were interpreted as supportive rather than independently causal. Negative urine toxicology, normal liver function markers, normal INR, albumin, and ammonia levels, and exclusion of relevant neurologic differentials substantially reduced alternative explanations. Following fluconazole 150 mg orally once daily for 21 days and a low-carbohydrate diet, repeat carbohydrate challenges every 3 weeks remained normal throughout 6 months of follow-up, and 6-month fungal cultures were negative. Beyond its diagnostic strength, the case is notable for its medicolegal and occupational consequence: a law-enforcement officer was dismissed for presumed alcoholism despite objective evidence of endogenous ethanol production. Early recognition of ABS may prevent stigma and inappropriate punitive attribution while enabling targeted treatment, safety counseling, and objective verification of remission.

## Data Availability

All relevant de-identified clinical data are included within the manuscript. Additional information may be available from the corresponding author upon reasonable request.

## Research registration unique identifying number (UIN)

Not applicable, as this is a case report.
